# Large Scale Triboelectric Nanogenerator and Self-Powered Flexible Sensor for Human Sleep Monitoring

**DOI:** 10.3390/s18061713

**Published:** 2018-05-25

**Authors:** Xiaoheng Ding, Hailin Cao, Xinghong Zhang, Mingyu Li, Yuntian Liu

**Affiliations:** 1 School of Material Science and Engineering, Harbin Institute of Technology, Harbin 150001, China; zhangxh@hit.edu.cn; 2 Shenzhen Academy of Aerospace Technology, Shenzhen 518000, China; caohl_cl@chinasaat.com (H.C.); mingyu.li@unswalumni.com (M.L.); 3 Department of Physics, University of Science and Technology of China, Hefei 230000, China; lyuntian@mail.ustc.edu.cn

**Keywords:** self-powered sensor, triboelectric, flexible sensor, sleeping monitoring

## Abstract

The triboelectric nanogenerator (TENG) and its application as a sensor is a popular research subject. There is demand for self-powered, flexible sensors with high sensitivity and high power-output for the next generation of consumer electronics. In this study, a 300 mm × 300 mm carbon nanotube (CNT)-doped porous PDMS film was successfully fabricated wherein the CNT influenced the micropore structure. A self-powered TENG tactile sensor was established according to triboelectric theory. The CNT-doped porous TENG showed a voltage output seven times higher than undoped porous TENG and 16 times higher than TENG with pure PDMS, respectively. The TENG successfully acquired human motion signals, breath signals, and heartbeat signals during a sleep monitoring experiment. The results presented here may provide an effective approach for fabricating large-scale and low-cost flexible TENG sensors.

## 1. Introduction

Advancements in consumer electronics and smart terminals have dramatically altered our daily lives and created urgent demand for new intelligent sensor technologies [1,2,3]. Previous research has made a great effort towards the development of high performance tactile sensors that have wide range sensitivity, a rapid response time, and portability. Tactile sensors are employed as smart devices in muscle motion monitors, intelligent watches, and other health monitors. These sensing technologies are usually based on various physical mechanisms including piezoresistivity, piezoelectricity, and capacitance. In particular, the piezoelectricity-based sensor is self-powered and is able to harvest electrical energy from kinetic energy and produce pulse signals during mechanical contact [4].

The triboelectric nanogenerator (TENG) is a relatively new technology which is able to harvest energy from motion in its surrounding environment. It can function as a nano-power source and tactile sensor simultaneously [3]. The TENG can convert mechanical energy into electricity through the contact-separation motion or relative sliding between two materials that have opposite electronegativities. TENGs may also be easily fabricated from a wide variety of materials and have superior flexibility to piezoelectric devices.

TENGs are based on the triboelectric effect and electrostatic induction which is affected by the material selection [5], device structure, and surface topography of the triboelectric contact surfaces. Surface topography plays an especially important role during the triboelectrification process [6,7,8]. Both the micro- and nano-scale surface morphological modifications result in a great increase in the specific surface area which hold a much more effective amount of charge and efficient electric potential [9]. Researchers have attempted many different approaches to enhance surface topography including plasma etching [10,11], photolithography [12,13], 3D printing [14], and chemical treatment [15]. Most of these approaches are very high in cost, however, and yield only small-sized products due to limited wafer or chamber size.

In this study, we designed a convenient method for the fabrication of large-scale, high-performance tactile sensors with induced micropores. The tactile sensors have fast response times and high sensitivity, with remarkable flexibility that may make them very well-suited to wearable electronics.

## 2. Experimental Procedures

**Figure 1:**
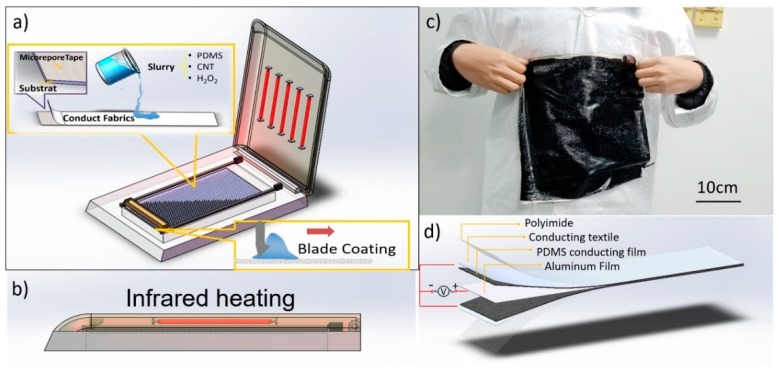
(**a**) Porous film fabrication process; (**b**) infrared heating chamber; (**c**) large-scale sample; (**d**) triboelectric nanogenerator (TENG) structure.

Premixed slurry was prepared with PDMS (Polydimethylsiloxane) precursor mixture (Dow Corning Sylgard 184), cross-linker and diluent at a weight ratio of PDMS to cross linker to diluent of 10:1:1; 1 wt % of CNT was added to the slurry and stirred until its color was uniform black. The slurry was then dispersed in an ultrasonic cell pulverizer at 80% power for 2 s of work followed by a 2 s break, for 5 min. Then, the slurry was cooled to room temperature and the ultrasonic dispersion step was repeated for 30 min. An 0.72 wt % H_2_O_2_ (30% H_2_O_2_ solution) was also added to the slurry and mixed with a magnetic stirring apparatus for 30 min. A schematic diagram of the porous film fabrication process is shown in Figure 1.

We first affixed the textile electrode on the template with 3M micropore tape, and then coated it with a 750-μm layer of premixed slurry on the electrode fabrics with a blade as shown in Figure 1a. The slurry was dried with infrared heating at 120 °C for 30 min as shown in Figure 1b. A foaming process occurred during the heating process; the self-decomposition of H_2_O_2_ was accelerated at an elevated temperature to create the porous structure. We finally peeled the film from the template and a 300 mm × 300 mm porous PDMS film with a textile electrode was fabricated successfully, as shown in Figure 1c.

We also made a TENG sample based on the triboelectric theory. We used aluminum as the triboelectric pair of PDMS because it is flexible, easy to clip, and inexpensive. Figure 1d shows the symmetrical structure of the TENG with aluminum and porous PDMS with textile electrodes and polyimide layers from the center outward.

The microstructures were imaged under a scanning electron microscope (Hitachi SU8010). The voltage outputs were measured using an oscilloscope (TELEDYNE LECROY WaveRunner 640Zi), where the adjustable impact load was supplied by a drop hammer tester. We also ran a fatigue test with a mechanical shaker under a pressure of 2.5 kPa and frequency of 5 Hz. The samples were also coated with a thin layer of copper on the surface without the electrode to measure the dielectric constant with an impedance analyzer (Agilent E4990A Precision Impedance Analyzer; Agilent Technologies, Santa Clara, CA, USA).

## 3. Results and Discussion

We prepared a TENG based on the electrostatic charges on the surfaces of two different materials as they physically contacted and separated. The charges on the surface increased until reaching a saturation state within several cycles of repeated contact and separation, which built up an electrostatic field in the gap between them. The electrostatic field drove electron flow through the external circuit leading to a buildup of free electrons in the electrodes.

The Maxwell displacement current for double layer contact and separate mode is expressed as follows [16]:

(1) JD=∂Dz∂t=∂σI(z,t)∂t=σcdzdtd1ε0ε1+d2ε0ε2[d1ε0ε1+d2ε0ε2+z]2+dσcdtzd1ε0ε1+d2ε0ε2+z,

where ε is the dielectric constant of materials, *d* is the thickness of materials, *z* is the distance between two dielectrics, and σ is the density of charges on the surface. According to Wang’s theory [16], the contact area between the triboelectric pair and the contact/separation speed determines the amount of charge and the open circuit voltage value [13,17,18,19].

**Figure 2:**
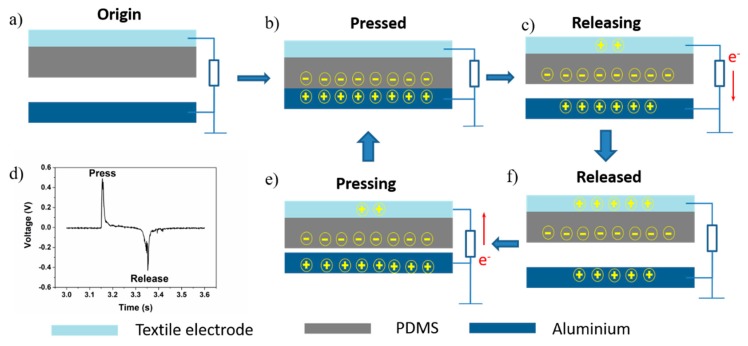
(**a**–**c**,**e**,**f**) TENG working principles; (**d**) press and release signal pulse.

The working principle of the TENG is evident in the coupling of contact charging and electrostatic induction [20]. Figure 2 shows a schematic diagram of the TENG. In its original state, the TENG system is balanced—there are no charge movements and no potential difference between two electrodes, as shown in Figure 1a. As shown in Figure 2b, when the PDMS and aluminum contact each other, charges transfer between their surfaces due to their different abilities to gain or lose charge. The PDMS carries negative charge while the aluminum carries the same amount of positive charge which keeps the TENG in the balanced state. When TENG starts to separate, the balance is broken due to the electrical potential difference. The PDMS can retain charges on its surface which remains unchanged, so a balanced state is rebuilt. The charges on the electrode surface transfer from the PDMS electrode to the aluminum as shown in Figure 2c,f. When the two surfaces are brought together again (Figure 2e), the electrical balance is broken and rebuilt again; charges transfer from the aluminum back to the PMDS electrode until balance is reached (Figure 2b).

**Figure 3:**
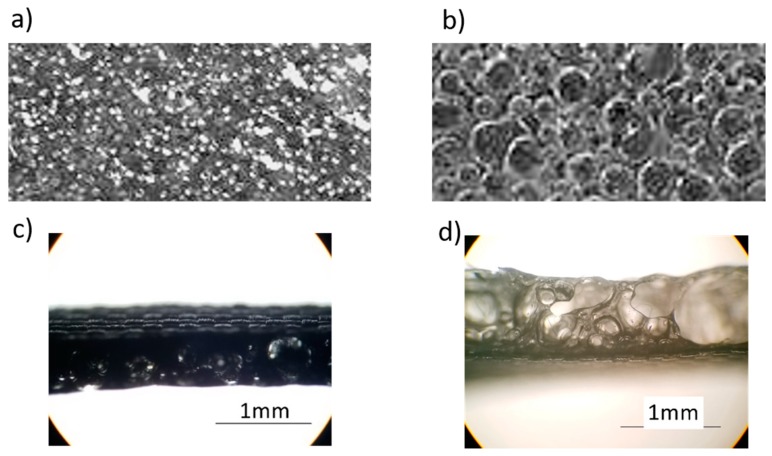
(**a**) Top surface of a carbon nanotube (CNT)-doped porous PDMS sample; (**b**) top surface of undoped porous PDMS sample; (**c**) cross sections of CNT-doped porous PDMS sample; (**d**) cross sections of undoped porous PDMS sample.

Figure 3 shows the foamy structure of the top surfaces and cross sections of the CNT-doped porous PDMS sample and undoped porous PDMS sample. These structures are related to the performance enhancement that we observed [21]. CNT-doped porous PDMS samples have closed bubble structures 0.2–0.5 mm in size, and the sample surface appears to be unbroken with uniform protuberance. The undoped porous PDMS sample has a broken bubble structure that creates a honeycomb-like appearance. The CNT material has a large length radius ratio which limits the growth of bubbles and reduces the foaming rate, so the PDMS curing process is faster than the foaming process, resulting in a small bubble size. The undoped PDMS film shows a honeycomb-like structure, having experienced a full bubble growth process including expansion, reciprocal squeezing, and breakage.

Figure 3c,d show that the porous PDMS and CNT-porous PDMS do have different thicknesses. The thicknesses of porous PDMS and CNT-doped porous PDMS were 0.89 and 0.67, respectively. It is difficult to precisely control the thickness of porous film with the current foaming process. According to Wang’s work [22], the contact-separation mode of TENG in this work is an example of the conductor-to-dielectric contact-mode of TENG. The output of this theoretical model is

$$V=-\frac{Q}{S\varepsilon_{0}}\left[\frac{d}{\varepsilon }+x\left(t\right)\right]+\frac{\sigma x(t)}{\varepsilon_{0}}$$

where *V* is the potential difference between two electrodes, *Q* is the charge transferred between electrodes that is driven by a potential difference at the minimum achievable charge reference state, *S* is the contact area of the triboelectric functional layers (aluminum layer and PMDS layer), *σ* is the charge that is generated in the triboelectric mechanism, *x* is the distance between the functional layer. The dielectric constant of the air in the gap is represented by *ε*_0_, and *ε*, *d* is the dielectric constant and thickness for the three types of PDMS layer. Hence, when *d* is far less than sample’s length of side (*S*^1/2^ in this study), and other parameters are kept the same, the output (*V*) has negligible difference for small differences in thickness.

**Figure 4:**
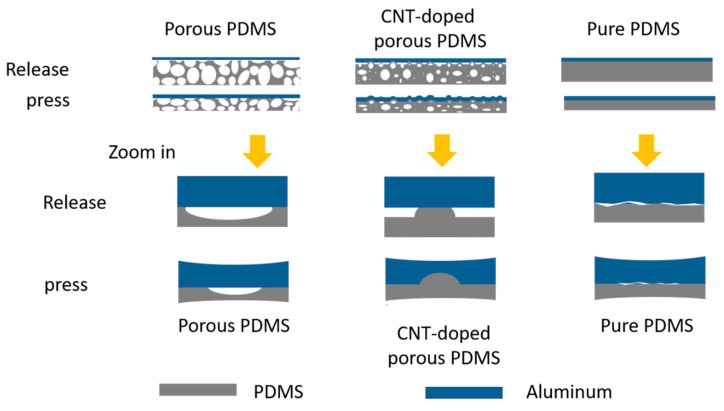
Schematic of contact-separation mode for different PDMS samples.

The actual contact/separation area markedly differed between the two samples, as shown in Figure 4. In this study, we aimed to build a device with high flexibility on a large-scale. Therefore, no spacers were used to fabricate the continuous space between functional layers. The functional layers of our sample appeared to be touching after encapsulation. Our TENG sensor has a different contact-separation process from traditional [20], arch type [23], and spring type [17] devices.

The porous PDMS is mainly composed of open cells with concave surfaces. The pore radius of curvature will be changed under pressure and lead to contact of the functional layer. CNT-doped porous PDMS mainly consists of closed cell structures with raised surfaces, and there are many irregular gaps are formed between the functional layers. In addition, microscopic contact defects exist between the dielectric and the electrode for pure PDMS without a porous structure, which is attributed to surface roughness, wrinkles and other factors.

In this study, different samples were tested with the same external circuit. Their sample thicknesses and dielectric constants were similar. The obvious differences in output between samples was due to their different effective contact-separation areas and contact separation speeds. Among these three type of samples, CNT-doped porous PDMS had the largest effective area due to the equivalent gap formed by the raised surface. Simultaneously, the elastic characteristic of the porous structure caused it to have a higher contact-separation speed during the action.

**Figure 5:**
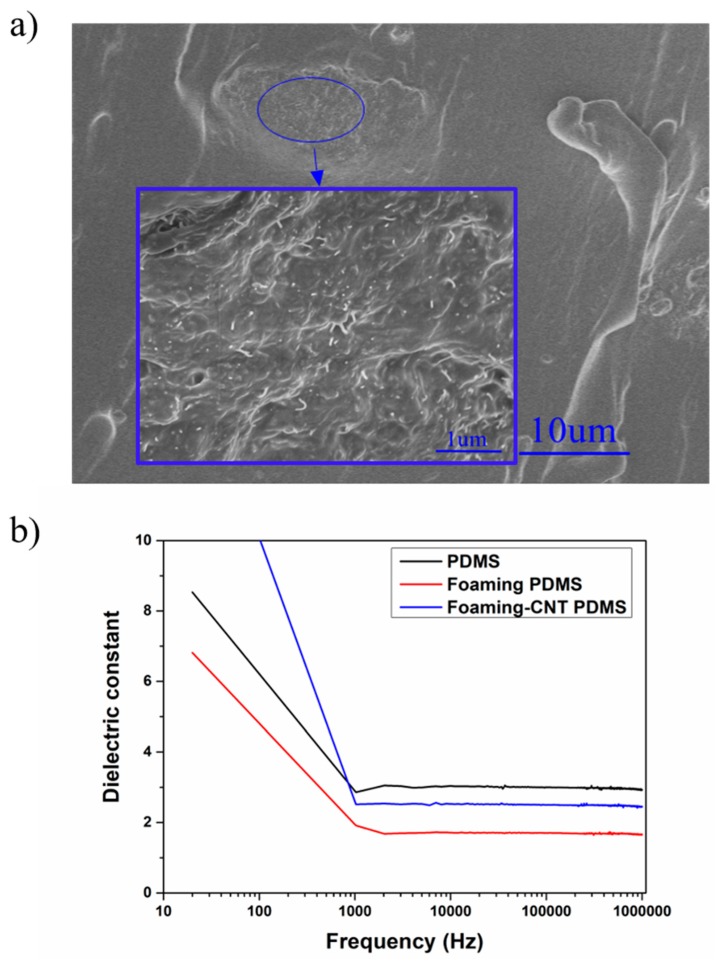
(**a**) SEM image of CNT-doped porous PDMS sample; (**b**) dielectric constants as a function of frequency for TENG with pure PDMS, TENG with undoped porous PDMS, and TENG with CNT-doped porous PDMS.

The influence of CNTs on the foaming structure can be ascribed to the curing process. We found that the foaming process was closely related to the gas production rate, PDMS curing time, and other mechanical properties in our sample (e.g., Young’s modulus). Polymer mechanical properties are often enhanced by the addition of nanoparticles. CNT material is commonly used to fabricate functional composites due to its unique characteristics including its large length radius ratio and excellent mechanical, electrical, and thermal properties [24,25,26,27]. The agglomeration of CNT in our PDMS sample exhibited random distribution on the macroscale, as shown in Figure 5a. The agglomeration of CNT created an “island-like” structure which had similar reinforcing behavior to the carbon black particle mechanism at the mesoscale [28,29]. At the micro-nano scale, the strengthening mechanism of CNT dominated the local reinforcement of the material. An isolated “island” does not create a conductive network, so the mechanical modification of PDMS did not affect its electrical properties and had negligible effect on the dielectric constant (Figure 5b). Sternstein’s group proposed that temporary (labile) bonding of chains to the nanofiller surface results in trapped entanglements, that have both near- and far-field effects on matrix chain motions. These trapped entanglements cause greatly enhanced non-Gaussian (Langevin) chain behavior that affects storage and loss moduli differently, resulting in very high reinforcement by nanofillers [30]. Huang’s group developed a Cohesion model based on van der Waals force interaction effect [31]. Follow-up works described the relationship between CNT and polymer matrix, and the relationship between interface stress and interface displacement from a physical point of view, they also developed a quantized method for measuring CNT reinforcement [32].

**Figure 6:**
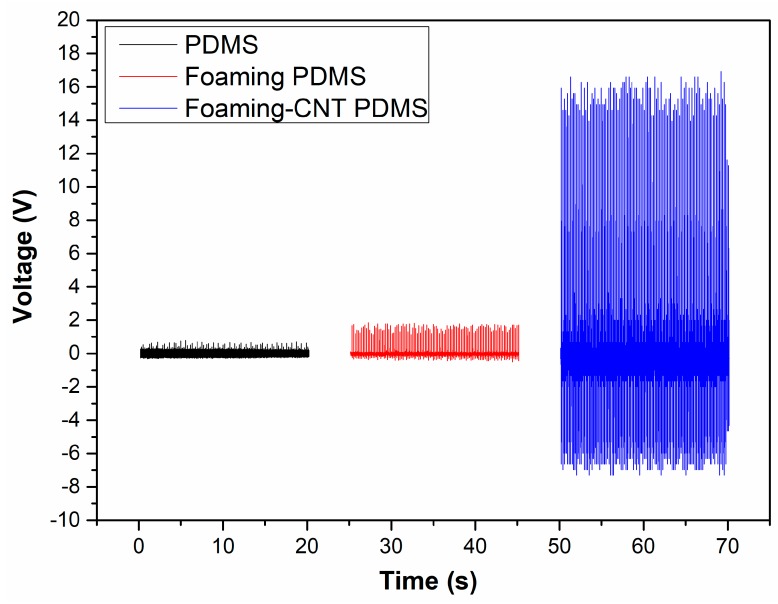
Voltage output patterns for TENG with pure PDMS, TENG with undoped porous PDMS, and TENG with CNT-doped porous PDMS.

All samples were tested with the same vibrator at the same speed and force. The voltage patterns were measured for 20 s as shown in Figure 6. The TENG with CNT-doped porous PDMS showed a much higher voltage output (16 V) while the TENG with pure PDMS and the TENG with undoped PDMS with a foaming structure had voltage outputs of 0.7 V and 2.2 V, respectively. The elevated voltage output is mainly attributable to the foaming structure and doping of CNT; the foaming structure allowed for faster contact speed and increased the contact area, and the addition of CNT reinforced the foaming structure to further enhance these qualities. According to Equation (1), fast contact speed and a large contact area result in a larger voltage output. The positive part was larger than the negative part due to the contact and separation speed as well—the contact speed was faster than the separation speed due to the foaming structure.

**Figure 7:**
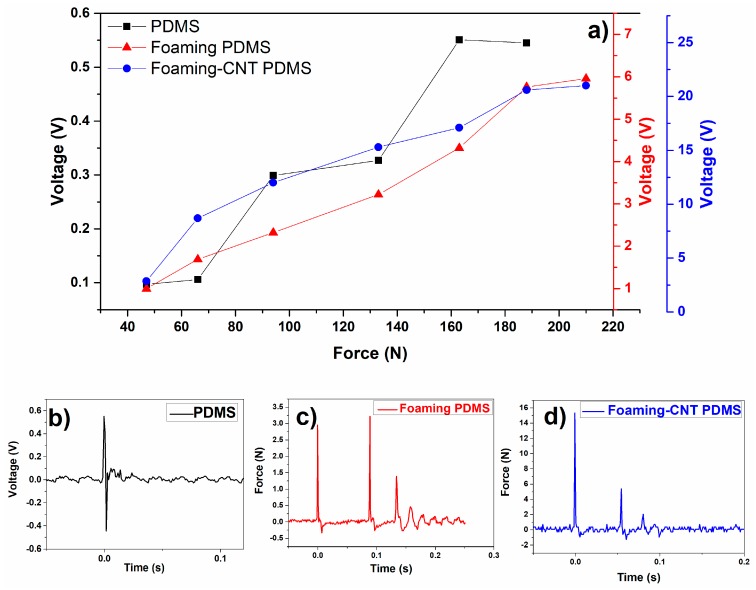
(**a**) Voltage outputs as a function of applied force; (**b**) voltage output pattern for TENG with pure PDMS; (**c**) voltage output pattern for TENG with undoped porous PDMS; (**d**) voltage output pattern for TENG with CNT-doped porous PDMS.

We also examined the response of TENG to different external forces by using a drop hammer tester to provide an adjustable load. Figure 7a shows the real-time profile of the voltage output and it corresponding applied load. The CNT-doped porous PDMS showed the highest voltage output, 21.5 V, when a load of 211N was applied. The peak voltage increased as the applied force increased in a linear manner in the CNT-doped sample, while the undoped sample showed step growth in its peak voltage.

Figure 7b–d show enlarged output voltage profiles of detailed patterns in the time domain. The samples appear to have had different response peaks after the test hammer dropped. Undoped pure PDMS only has one positive and negative impulse wave couple. However, the two foaming PDMS samples show several impulse pairs due to the porous elastic structure. Only the CNT-doped porous samples show the ideal damping process of hammer rebound; the undoped foaming PDMS sample shows an irregular damping process that is attributable to its irregular surface structure. In short, the porous TENG is more resilient.

**Figure 8:**
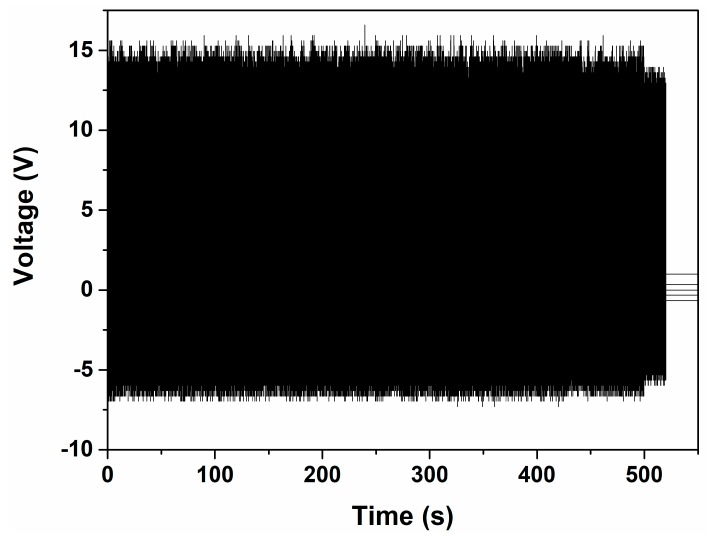
Fatigue tests on CNT-doped porous TENG.

We also investigated the stability of the CNT-doped porous TENG sample with a mechanical shaker. A pressure of 2.5 kPa and frequency of 5 Hz was applied for 5 h. Figure 8 shows the last 500 s of recorded voltage output, which exhibits negligible changes after 90,000 cycles of fatigue testing. The CNT-doped porous TENG appears to have very high stability and durability.

Sleep monitoring systems are useful for the early detection and prevention of disorders such as sleep apnea syndrome and cardiovascular diseases. They provide informative readouts of human health indicators including heart rate, muscle movement, and breath.

**Figure 9:**
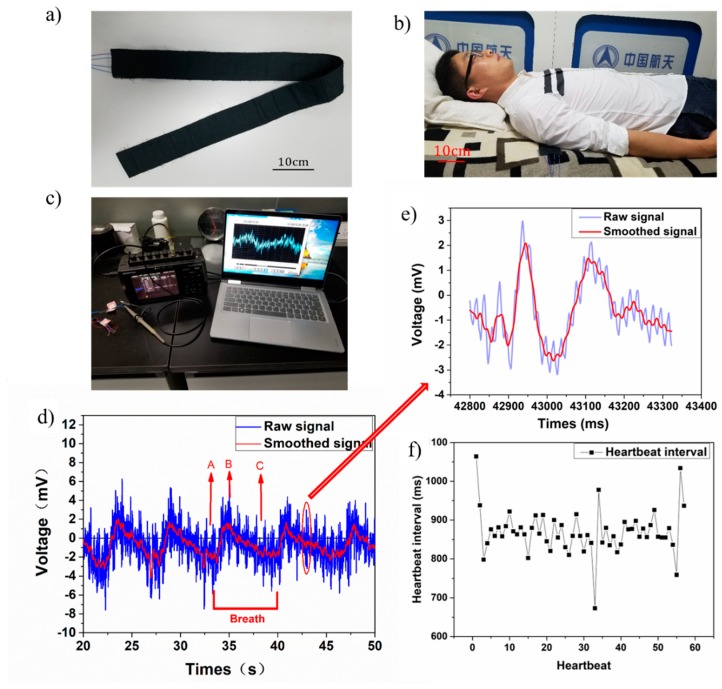
(**a**) Sleep monitoring belt; (**b**) user sleeping with monitoring belt; (**c**) measurement system setup; (**d**) real-time voltage output of breath and heartbeat; (**e**) enlarged heartbeat signal; (**f**) heartbeat interval distribution.

In this study, we developed a sleep monitoring belt with the proposed CNT-doped porous TENG, as shown in Figure 9a. This belt consists of two TENG parts in a parallel configuration with two independent signal passageways that catch independent signals at different positions. As shown in Figure 9b, the belt is placed under the chest to monitor the user’s breath and heartbeat signals during sleep. Figure 9c shows that the measurement system was used to capture the breath and heartbeat signals. Figure 9d shows the ballistocardiogram of a real-time voltage output as the user slept for 30 s using the prototype belt. The raw signal clearly shows a breath signal with a low frequency and a heartbeat signal with a higher frequency than the breath signal. The real-time recorded heartbeat signal shows an uptick upon the breathing signal. The tester had a regular heartbeat rate of around 70 beats per cycle. The smoothed signal revealed our tester’s breath signal after data processing, as a typical slow wave shape indicative of the breathing signal. Our tester breathed about five cycles in a prone position over the course of 30 s. The breathing signals showed two distinct stages: point A to point B represents the inhalation process and point B to point C represents the exhalation process. Inhalation processes are shorter than exhalation processes—the former takes our tester approximately 2 s and the latter approximately 5 s. We also showed an enlarged single heartbeat signal in Figure 9e. The enlarged image shows more detailed heartbeat characteristics use both raw signal and smoothed signal. The smoothed signal had two characteristic peaks in each heartbeat cycle with a 0.12 s difference in the time domain. These two peaks likely represent the heart pumping venous blood and arterial blood, respectively.

The heartbeat signal was processed and plotted into the diagram shown in Figure 9f. The heartbeat intervals and distribution are indicative of heart rate variability and could, in practice, be assessed to determine any anomalies. The degree of damage to the coronary artery in heart disease patients is related to heart rate variability [33]. A monitor like this one also could be utilized to detect chronic cardiac failure [34]. In this study, the tester’s heart rate approached 70 beats per minute and exhibited reasonable heart rate variability.

## 4. Conclusions

In this study, we successfully developed a self-powered tactile sensor with CNT-doped porous TENG. The TENG sensor showed several notable advantages including a fast response, high sensitivity, and excellent durability. It also has the ability to be produced at large scale and low cost. The sleep monitoring belt designed using the proposed sensor provided accurate information about real-time breath and heartbeat, suggesting it has potential application as a health monitor for disease diagnosis and emergency prevention.
